# Moso bamboo invasion alters soil microbial nutrient limitation by modifying plant diversity and soil nutrient cycling in subtropical forest

**DOI:** 10.3389/fpls.2025.1549018

**Published:** 2025-10-30

**Authors:** Jiannan Wang, Shihao Wen, Weiqiang Liu, Zitian Hao, Xiuping Wu, Sili Peng, Zhiwei Ge, Xiaoyue Lin, Lingfeng Mao

**Affiliations:** ^1^ Laboratory of Biodiversity and Conservation, Co-Innovation Center for Sustainable Forestry in Southern China, College of Ecology and Environment, Nanjing Forestry University, Nanjing, China; ^2^ Co-Innovation Center for Sustainable Forestry in Southern China, College of Ecology and the Environment, Bamboo Research Institute, Nanjing Forestry University, Nanjing, China; ^3^ Forestry Bureau of Longyou County, Quzhou, China

**Keywords:** Moso bamboo invasion, *Phyllostachys edulis*, soil microbial nutrient limitation, extracellular enzyme activity, plant diversity, subtropical forest

## Abstract

**Introduction:**

In recent years, increasing management costs and declining market prices for Moso bamboo (Phyllostachys edulis) have led to the abandonment of many Moso bamboo forests, resulting in their gradual encroachment into neighboring broadleaf forests—a phenomenon that continues to intensify in subtropical regions of China. Moso bamboo invasion has significant impacts on ecosystem processes and functions; however, its effects on soil microbial nutrient limitations remain unclear.

**Methods:**

Here, we employed a space-for-time substitution by selecting plots representing four stages of Moso bamboo invasion and measuring plant community diversity, soil physicochemical properties, and extracellular enzyme activities related to carbon, nitrogen, and phosphorus cycling.

**Results:**

Results showed that bamboo invasion reduced overstory (tree layer) diversity but increased diversity in the shrub and herb layers. Soil total organic carbon (TOC), total nitrogen (TN), available phosphorus (AP), and available potassium (AK) all decreased significantly with increasing invasion intensity. In contrast, soil pH and the activities of β-1, 4-glucosidase (BG), N-acetyl-β-glucosaminidase + leucine aminopeptidase (NAG+LAP), and acid phosphatase (ACP) increased along the invasion gradient. Throughout the invasion process, microbial C limitation intensified (longer vector length), whereas P limitation was partially alleviated (vector angle decreased). These shifts in microbial nutrient limitation were closely related to changes in soil nutrient content and plant diversity.

**Discussion:**

Our findings indicate that Moso bamboo invasion alters soil microbial nutrient acquisition strategies by reducing carbon inputs (enhancing C limitation) and relatively relaxing P limitation. These microbial nutrient limitation changes correspond with reduced tree litter and increased shrub/herb presence. The study provides new insights into invasion ecology and offers guidance for managing Moso bamboo spread in subtropical forests.

## 1 Introduction

Plant invasions that drive forest succession can significantly alter primary productivity, organic matter decomposition, and nutrient cycling in ecosystems (Ahmad et al., 2020). These changes can extend to geomorphological and hydrological processes, ultimately leading to biodiversity loss and reduced forest ecosystem stability (Stanek and Stefanowicz, 2019). With the intensifying effects of global climate change and human activities, the proliferation of invasive plants has become a critical environmental challenge worldwide (Rai and Singh, 2020). Invasion ecology theory suggests that the impact of an invasive species depends not only on the invader’s traits but also on the vulnerability of the invaded ecosystem (Li et al., 2024). Classic hypotheses such as Elton’s “diversity-invasibility” hypothesis posit that species-rich communities are more resistant to invasion (Elton, 1958). However, empirical studies show mixed results, and in some cases invasions occur in diverse ecosystems. These discrepancies indicate that factors like specific functional traits of invaders and belowground interactions can modulate invasion outcomes (Ernst et al., 2022; Li et al., 2024).

Soil microorganisms are pivotal mediators of the impacts of plant invasions on ecosystems. They drive key biogeochemical processes (e.g., decomposition and nutrient mineralization) and influence plant growth through mutualistic relationships and pathogen dynamics (Sardar et al., 2023). An invader that alters soil microbial communities can therefore change nutrient cycling in ways that feed back to plant community composition (Zhang et al., 2023). Previous work has shown that some invasive plants can increase microbial activity or biomass by providing labile carbon, whereas others can suppress soil microbes by introducing recalcitrant litter or allelopathic compounds. In either case, linking plant invasion to shifts in soil microbial nutrient limitations is crucial for understanding the full ecosystem consequences (Zhang et al., 2019). Here, nutrient limitation refers to the element (C, N, or P) that most strongly constrains microbial growth and activity. Changes in microbial nutrient limitation status could signal shifts in ecosystem nutrient availability and cycling efficiency (Sinsabaugh and Follstad Shah, 2012).

Ecological stoichiometry provides a useful framework to study microbial nutrient limitations in invaded soils. Soil extracellular enzyme activities reflect microbial investment in acquiring particular nutrients (C, N, or P) and thus can serve as proxies for microbial nutrient demand (Yang et al., 2023). By measuring the ratios of C-, N-, and P-acquiring enzyme activities, one can infer which nutrient is relatively limiting to the microbes. For example, if microbes greatly increase C-degrading enzyme activity relative to N- or P-degrading enzymes, it suggests carbon is limiting (i.e., microbes are “foraging” for C) (Li et al., 2024). Researchers have developed enzyme stoichiometry metrics and vector analysis techniques to quantify these limitations. In this approach, the vector length represents overall microbial resource limitation (longer vectors indicate stronger C limitation) and the vector angle indicates the balance of N versus P limitation (angles<45° suggest closer to P limitation, >45° suggest closer to N limitation) (Li et al., 2023). Such enzyme-based indicators respond faster than bulk soil nutrient measures, providing an early signal of changing nutrient constraints on microbes (Moorhead et al., 2016). They have been applied in various ecosystems to diagnose nutrient limitation and have shown sensitivity to factors like climate, soil type, and vegetation changes.

Moso bamboo (*Phyllostachys edulis*), a woody bamboo species in the subfamily Bambusoideae (Poaceae), is capable of exhibiting invasive behavior and is widely distributed across East and Southeast Asia (Yang et al., 2015; Xu et al., 2020). Its extensive rhizome network and clonal reproductive ability facilitate rapid growth and expansion into adjacent forest areas. Through superior resource competition, Moso bamboo can suppress other tree species, ultimately transforming mixed forests into bamboo-dominated stands or even pure bamboo forests (Chen et al., 2024). This phenomenon of bamboo overabundance is also known as a native plant invasion (Ouyang et al., 2022), and it has become increasingly common throughout southern China’s bamboo distribution zones (Liu et al., 2021). During the 1860s and 1870s, substantial areas of subtropical evergreen broadleaf forest were cleared and converted to managed Moso bamboo plantations due to the high economic value of bamboo (Qi et al., 2022). In recent years, however, many bamboo plantations have been abandoned due to rising management costs and falling bamboo timber prices, leading to the uncontrolled expansion of bamboo into neighboring broadleaf forests (Chen et al., 2022).

There is growing evidence that such bamboo invasions alter aboveground community structure and soil conditions. Studies have reported that Moso bamboo expansion simplifies forest structure and reduces native plant diversity (Chen et al., 2022; Lacerda and Kellermann, 2019). Soil organic carbon and total nitrogen often decline in bamboo-invaded stands, likely due to faster litter decomposition and rapid uptake by bamboo (Liu et al., 2019). However, other soil nutrients can show different trends: for instance, one study found increased available N and P in bamboo-invaded soils, possibly due to faster litter turnover (Li et al., 2017). These conflicting findings suggest that bamboo’s impact on soil nutrients may depend on site-specific factors or invasion stage. Previous research indicates that Moso bamboo invasion can change plant diversity, soil chemistry, and microbial communities. Yet there is a knowledge gap in linking these changes to shifts in microbial nutrient limitation – a key aspect of nutrient cycling. Understanding this link is important for both ecological theory and practical forest management. Theoretically, it addresses how a dominant native invader can act as a driver of ecosystem functional change (sensu the “driver-passenger” model of invasions). Practically, it can inform predictions of long-term soil fertility under bamboo expansion and guide interventions to maintain ecosystem health.

In this study, we selected a typical site where a Moso bamboo forest is actively expanding into an evergreen broadleaf forest. Using a space-for-time substitution approach, we simulated different stages of Moso bamboo invasion by quantifying invasion intensity based on the percentage of bamboo stems in sample plots. We measured species diversity, soil physicochemical properties, enzyme activities, and soil enzyme stoichiometry across these invasion stages. We hypothesized that: (1) Moso bamboo invasion would significantly alter soil enzyme stoichiometry, reflecting a shift in microbial nutrient limitations—we expected increased microbial C limitation (due to reduced soil C inputs from broadleaf litter) and decreased P limitation (due to bamboo’s high N demand possibly rendering P relatively less limiting); and (2) these changes in microbial nutrient limitation would correlate with changes in plant diversity and soil nutrient content. The retention of diverse understory species and soil nutrient capital might mitigate emerging nutrient limitations. Our study aims to deepen understanding of ecosystem responses to Moso bamboo invasion by linking aboveground and belowground changes. By integrating invasion ecology and microbial ecology, we provide insight into the mechanisms by which a native species turning invasive can alter nutrient cycling. The findings are expected to inform forest management and policy by identifying key indicators of soil degradation during bamboo invasion. This knowledge can help in developing targeted strategies to control bamboo spread and maintain soil health in subtropical forests.

## 2 Materials and methods

### 2.1 Study area overview and sample collection

This research was conducted in Xikou Town (28°53′N, 119°14′E), located in Longyou County, Quzhou City, Zhejiang Province, China. The area experiences a subtropical monsoon climate with a mean annual temperature of 17.3 °C and average annual precipitation of 1,800 mm. The predominant soil type is red soil derived from granite, and the regional vegetation is dominated by evergreen broadleaf forests and Moso bamboo stands. During the 1860s and 1870s, large expanses of native broadleaf forests were cleared for Moso bamboo plantations due to the significant economic value of bamboo products. However, in recent years, many of these bamboo forests have been abandoned because of rising management costs and falling bamboo wood prices, enabling them to encroach into neighboring broadleaf forests.

In September 2023, we established four transects along the front of bamboo expansion where Moso bamboo was encroaching into evergreen broad-leaved forest. We employed a space-for-time substitution to capture different stages of bamboo invasion, quantifying the degree of invasion based on the percentage of Moso bamboo stems relative to total tree stems in each plot. Each transect encompassed four forest types representing increasing invasion: broad-leaved forest (BF; 0% bamboo), low-mixture forest (LM; 20–40% bamboo), high-mixture forest (HM; 60–80% bamboo), and Moso bamboo forest (MB; ~100% bamboo). The space-for-time approach assumes that the BF plots represent the pre-invasion state of the LM/HM/MB plots. Although this method cannot perfectly replicate a true time series, it is a practical approach widely used to infer successional and invasion impacts (Pickett, 1989; Suzuki and Hirao, 2018). We selected plots with similar elevation (300–350 m, gentle slopes with comparable aspect), soil type, and slope to minimize confounding site differences. Four 20 m × 20 m plots were established for each invasion type, totaling 16 plots. Within each 20×20 m plot, five 5×5 m subplots were placed at the four corners and center to survey the shrub layer. Additionally, within each subplot, five 1×1 m quadrats were established for the herb layer survey. We conducted comprehensive vegetation surveys in all plots (trees, shrubs, and herbs) and collected soil samples. To obtain representative soil samples, ten soil cores (0–20 cm depth) were collected from each plot following a “2-3-2-3” sampling pattern and combined to form one composite sample per plot. Each composite soil sample was sieved through a 2 mm mesh to remove stones and plant debris. A portion of the fresh sample was stored at 4 °C for enzyme activity analysis, and the remainder was air-dried at room temperature for physicochemical analyses.

### 2.2 Species diversity assessment

Species diversity was evaluated by calculating the Margalef richness index (R), Simpson index (D), Shannon-Wiener index (H′), and Pielou’s evenness index (E) for each plot. The formulas are as follows:


$$\text{Margalef index of species richness}:\text{R}=\frac{\left(\text{S}-1\right)}{\ln\text{N}}\;$$

$$\text{Simpson index }:\text{D}=\frac{\sum_{i=1}^{S}{\text{n}}_{i}\left({\text{n}}_{i}-1\right)}{\text{N}\left(\text{N}-1\right)}$$

$$\text{Shannon}-\text{Wiener index }:{\text{H}}^{\prime }=-\sum_{i=1}^{S}\text{Pi}\ln{\text{P}}_{i}$$

$$\text{Pielou evenness index }:\text{E}=\frac{{\text{H}}^{\prime }}{\ln\text{S}}$$

Where, for different community layers, **S** is the total number of species in the layer, **N** is the total number of individuals of all species in the layer, **n_i_** is the number of individuals of species *i* in the layer, and **P_i_** is the proportion of individuals of species *i* relative to the total number of individuals in the layer.

### 2.3 Soil physicochemical properties and enzyme activity measurement

Soil pH was determined using a potentiometric method with a soil-to-water ratio of 1:2.5. Soil moisture content was determined by the drying method. Total organic carbon (TOC) and total nitrogen (TN) were measured using an elemental analyzer (Elementar Vario EL cube, Germany). Total phosphorus (TP) was determined by sulfuric acid-perchloric acid digestion followed by molybdenum-antimony colorimetry. Alkaline nitrogen (AN) was measured using alkali hydrolysis diffusion method, and available phosphorus (AP) was extracted with 0.5 M NaHCO_3_ solution and determined by molybdenum-antimony colorimetry (Gai et al., 2021). Key enzymes reflecting soil carbon, nitrogen, and phosphorus cycling were selected: β-1,4-glucosidase (BG, EC 3.2.1.21), N-acetyl-β-glucosaminidase (NAG, EC 3.2.1.14), leucine aminopeptidase (LAP, EC 3.4.11.1), and acid phosphatase (ACP, EC 3.1.3.2). Enzyme activities were measured using a fluorometric method (Zhang et al., 2023).

### 2.4 Quantification of microbial nutrient limitation

We performed standardized major axis (SMA) regression on log-transformed enzyme activities to assess microbial nutrient investment relationships. This analysis tests the slope of the relationship between two variables without designating one as independent, accounting for variability in both (Warton et al., 2006; Smith, 2009). We ran SMA regressions for BG vs. (NAG+LAP), BG vs. ACP, and (NAG+LAP) vs. ACP across all plots. We then tested whether the SMA slopes differed significantly from 1 (the 1:1 line expectation) using line equality tests. A slope significantly greater or less than 1 indicates an imbalance in enzyme investment between those nutrient acquisition activities. Based on the SMA results, we further explored the likely nutrient limitations indicated by any imbalances.

Three common methods exist for evaluating soil microorganism nutrient limitation. The first method involves calculating enzymatic stoichiometric ratios for C, N, and P acquisition, and synthesizing results with soil C, N, P stoichiometry. The microbial enzyme stoichiometric ratios are calculated using these formulas.


$$\text{C}:{\text{N}}_{EEA}=\ln\left(\text{BG}\right):\ln\left(\text{NAG}+\text{LAP}\right)$$

$$\text{C}:{\text{P}}_{EEA}=\ln\left(\text{BG}\right):\ln\text{ACP}$$

$$\text{N}:{\text{P}}_{EEA}=\ln\left(\text{NAG}+\text{LAP}\right):\ln\text{ACP}$$

The second and third methods are enzyme ratio scatter plot and enzyme vector analysis. Sinsabaugh et al. (2008) and Hill et al. (2012) employ the N:P_EEA_ versus C:N_EEA_ as the X and Y axes, respectively. Using the value 1 (x=1, y=1) as the reference line, the resulting plot divides into four quadrants that indicate different limitation patterns: N limitation, P limitation, C and N co-limitation, and C and P co-limitation. Microbial nutrient limitation can also be assessed through enzyme vector analysis, where greater vector length indicates higher microbial carbon limitation; vector angles >45° and<45° represent N limitation and P limitation respectively, with greater angular deviation from 45° indicating higher N or P limitation (Moorhead et al., 2016). Vector length and angle are calculated using the following formulas:


$$\text{Vectorlength}=\text{SQRT }({\left(\text{lnBG}/\text{lnACP}\right)}^{2}+\left[\text{lnBG}/\text{ln}\left(\text{NAG}+\text{LAP}\right){]}^{2}\right)$$

Vector Angle=Degrees [ATAN2 [lnBG/lnACP, InBG/ln(NAG+LAP)]

### 2.5 Statistical analysis

One-way ANOVA was used to examine the effects of Moso bamboo expansion on species diversity, soil physicochemical properties, and enzyme activities (P ≤ 0.05). Tukey’s multiple comparison test was performed to analyze differences among Moso bamboo expansion gradients. Standardized major axis (SMA) regression analysis of soil C, N, and P-acquiring enzyme activities was conducted to determine whether microorganism nutrient limitations were present. Subsequently, enzyme ratio scatter plot and soil enzyme stoichiometry vector length and angle plots were used to further examine microbial nutrient limitations and their changes.

To identify the driving factors behind microbial nutrient limitations during Moso bamboo expansion, correlation analyses were first conducted between soil properties and soil enzyme activities to explore the direct relationships between enzyme activities and their corresponding soil substrates. Next, general linear regression analyses were performed between species diversity indices, soil physicochemical characteristics, and vector length and angle. Based on the regression results, significantly correlated indicators were selected and visualized. Finally, to determine the most influential factor affecting microbial nutrients during Moso bamboo expansion, a generalized linear mixed-effects model (GLMM) was constructed as follows:

lmer(Vector length/Vector Angle ~ Significantly correlated indicators + Bamboo expansion gradient + (1|Plot)). The glmm.hp package in R was used to quantify the relative contributions of these predictors. glmm.hp package enable hierarchical partitioning to calculates the variable importance from all subset models, leading to an unordered assessment of importance (Lai et al). All plotting and analyses were performed using R version 4.4.2.

## 3 Results

### 3.1 Plant community diversity

During Moso bamboo’s expansion into broadleaf forests, tree layer species diversity decreased significantly (P ≤ 0.05), as reflected in both species richness and diversity indices (Shannon-Wiener, Simpson, Margalef, and Pielou) (**Table 1**). All indices follow the pattern of BF > LM > HM > MB (**Table 1**). In the early invasion stage, the decline in species diversity was moderate, with no significant differences in indices between BF and LM. During the late invasion stage, there were significant differences in species diversity indices among MB, HM, and the pre-invasion stage (BF, LM) (**Table 1**). For the shrub layer, the Pielou, Simpson, and Shannon-Wiener indices showed no significant differences across varying levels of invasion, with the Shannon-Wiener index showing an increasing trend with Moso bamboo invasion. In contrast, the number of species and the Margalef index increased significantly (P ≤ 0.05), indicating higher species diversity in MB and HM compared to LM and BF (**Table 1**). The herb layer showed increased species diversity with Moso bamboo invasion, reflected in the number of species, Shannon-Wiener index, Simpson index, and Margalef index (**Table 1**). During the early stages of Moso bamboo invasion, species diversity did not increase significantly (P ≤ 0.05), and there were no significant differences in the indicator values between BF and LM. As the level of invasion increased, all indicators showed an upward trend. In the late stages of invasion, the indicator values for MB were significantly (P ≤ 0.05) higher than those of the other groups (**Table 1**).

**Table 1:** Changes in soil physicochemical properties of Moso bamboo invasion process.

Soil properties	BF	LM	HM	MB
pH	4.39 ± 0.16a	4.62 ± 0.14ab	4.76 ± 0.12b	5.24 ± 0.1c
TOC (g/kg^-1^)	30.74 ± 3.49c	24.78 ± 2.11b	19.47 ± 0.66a	17.07 ± 1.62a
TN (g/kg^-1^)	2.13 ± 0.28c	1.95 ± 0.11bc	1.61 ± 0.13ab	1.48 ± 0.19a
TP (g/kg^-1^)	0.22 ± 0.07	0.23 ± 0.06	0.23 ± 0.03	0.23 ± 0.02
AN (g/kg^-1^)	137.29 ± 9.15a	183.07 ± 19.76b	177.41 ± 13.4b	205.5 ± 8.11b
AP (g/kg^-1^)	6.25 ± 1.46	6.38 ± 1.18	6.38 ± 1.98	5.49 ± 1.23
AK (g/kg^-1^)	132.36 ± 10.32c	135.05 ± 10.25c	108.51 ± 8.01b	76.78 ± 10.69a
C:N	14.67 ± 2.52	12.75 ± 1.24	12.15 ± 0.58	11.62 ± 1.22
C:P	146.84 ± 28.3b	111 ± 21.3ab	84.46 ± 12.26a	75.46 ± 10.44a
N:P	10.47 ± 3.77	8.81 ± 2.19	7 ± 1.25	6.55 ± 1.08

TOC, total organic carbon; TN, total nitrogen; TP, total phosphorus; AN, available nitrogen; AP, available phosphorus; AK, available potassium; C:N, TOC and TN ratio; C:P, TOC and TP ratio; N:P, TN and TP ratio. Different lowercase letters in the same rows indicate significant difference at p ≤ 0.05 (Tukey’s HSD, mean ± SD, N = 4).

### 3.2 Soil properties

Soil TOC, TN, AP, and AK contents decreased with increasing Moso bamboo invasion intensity. Similarly, C:N, C:P, and N:P stoichiometric ratios also decreased with invasion intensity(**Table 2**). During the early stages of Moso bamboo invasion, TOC content in BF was significantly (P ≤ 0.05) higher than other groups, while LM showed significantly (P ≤ 0.05) higher TOC than HM and MB (**Table 2**). TN and AK contents showed similar trends, with notable differences between early and late invasion stages. The contents of TN and AK during the early stages of invasion (BF and LM groups) were significantly (P ≤ 0.05) higher than those in the late stages of invasion (HM and MB groups), while MB showed significantly (P ≤ 0.05) lower AK content than all other groups (**Table 2**). AP content showed an increasing trend, although no significant differences were observed among the groups. In contrast, AN content increased significantly (P ≤ 0.05), with MB showing significantly (P ≤ 0.05) higher AN content than LM, HM, and BF (**Table 2**). All soil samples in this study were weakly acidic, and pH values steadily increased with Moso bamboo invasion. The pH in the early invasion stage (BF) was significantly (P ≤ 0.05) lower than in the other groups (**Table 2**). Regarding nutrient stoichiometric ratios, the C:N, C:P, and N:P ratios all decreased to some extent with increasing Moso bamboo invasion intensity. In the early invasion stage (BF group), the C:N, C:P, and N:P ratios were significantly (P ≤ 0.05) higher than those in the late invasion stages (HM and MB groups) (**Table 2**).

**Table 2:** Changes in species diversity of each layer in the Moso bamboo invasion process.

Layle	treat	Rich	Margalef	Pielou	Simpson	Shannon
tree	BF	16 ± 2.31a	3.17 ± 0.34a	0.79 ± 0.08a	0.83 ± 0.08a	2.2 ± 0.34a
	LM	16 ± 1.83a	3.02 ± 0.29a	0.75 ± 0.05a	0.82 ± 0.03a	2.09 ± 0.18a
	HM	11.5 ± 1.73b	2.19 ± 0.3b	0.51 ± 0.05b	0.51 ± 0.06b	1.23 ± 0.11b
	MB	1c	0c	0c	0c	0c
shrub	BF	25.75 ± 2.22b	5.02 ± 0.36ab	0.84 ± 0.05	0.91 ± 0.03	2.73 ± 0.18
	LM	25.75 ± 2.5b	4.84 ± 0.56b	0.85 ± 0.01	0.91 ± 0.02	2.76 ± 0.13
	HM	31.25 ± 3.77ab	5.62 ± 0.61ab	0.84 ± 0.02	0.92 ± 0.02	2.91 ± 0.17
	MB	35 ± 2a	5.95 ± 0.45a	0.82 ± 0.03	0.92 ± 0.02	2.93 ± 0.15
herb	BF	2.25 ± 0.5b	0.4 ± 0.13b	0.51 ± 0.19b	0.23 ± 0.11c	0.4 ± 0.16c
	LM	2.25 ± 0.5b	0.6 ± 0.26b	0.84 ± 0.22ab	0.45 ± 0.19bc	0.68 ± 0.3bc
	HM	3.25 ± 0.96b	0.89 ± 0.26b	0.88 ± 0.13a	0.58 ± 0.11ab	0.98 ± 0.27b
	MB	13.25 ± 2.22a	2.57 ± 0.33a	0.74 ± 0.08ab	0.76 ± 0.07a	1.9 ± 0.31a

Different lowercase letters in the same column indicate significant differences among forest types at the same layer at p ≤ 0.05 (Tukey’s HSD, mean ± SD, N = 4).

### 3.3 Soil extracellular enzyme activity and stoichiometry

**Figure 1:**
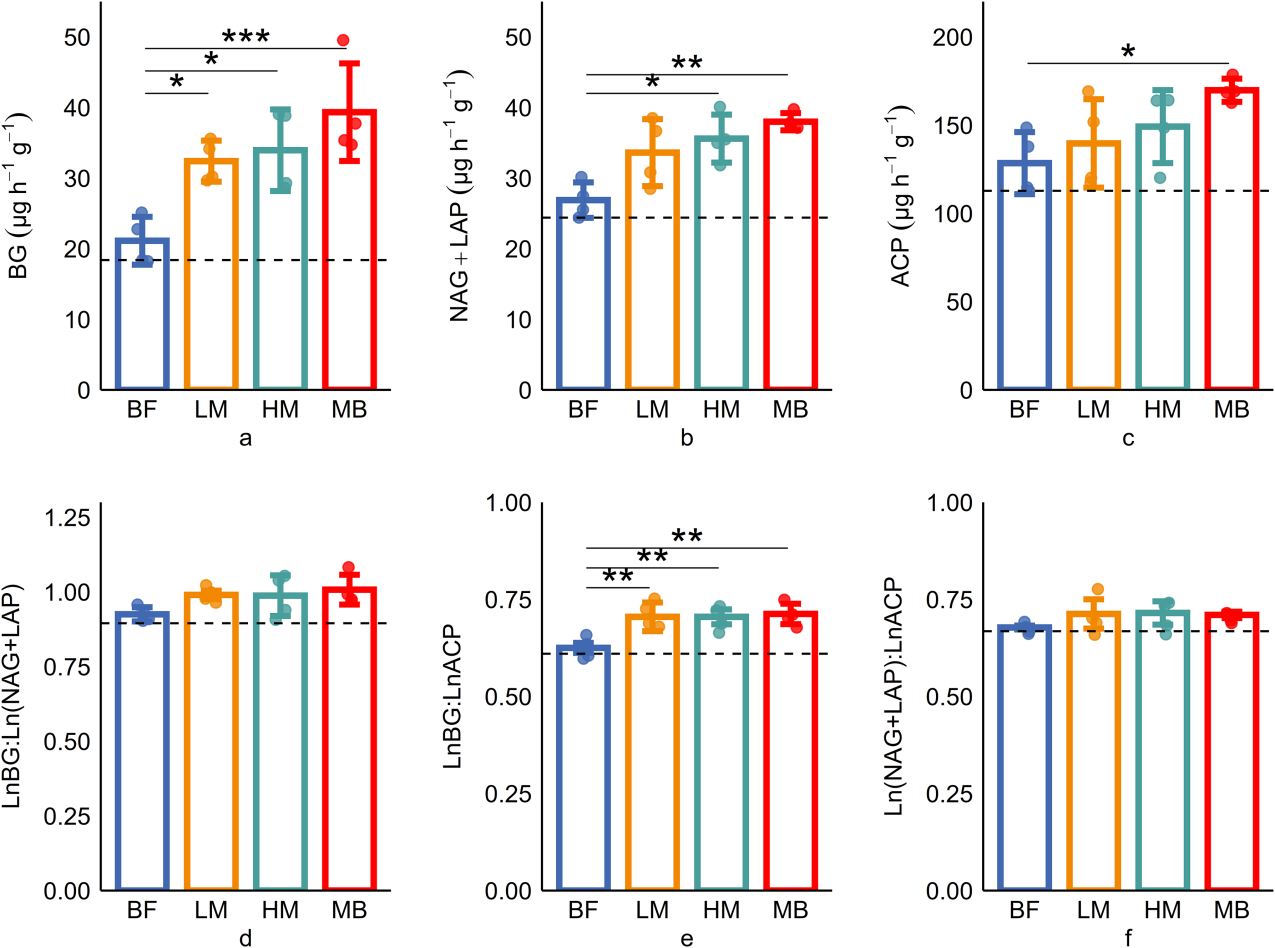
Soil enzyme activities **(a–c)** and stoichiometries **(d–f)** response to Moso bamboo invasion. BG, β-1,4glucosidase; NAG, β-1,4-N-acetylglucosaminidase; LAP, L-leucine aminopeptidase; ACP, acid phosphatase; C:N_EEA_, ln(BG): ln(NAG + LAP); C:P_EEA_, ln(BG): ln(ACP); N:P_EEA_, ln(NAG + LAP): ln (ACP). Data were presented as mean ± SD (N = 4, * p ≤ 0.05, ** p ≤ 0.01, *** p ≤ 0.001).

**Figure 2:**
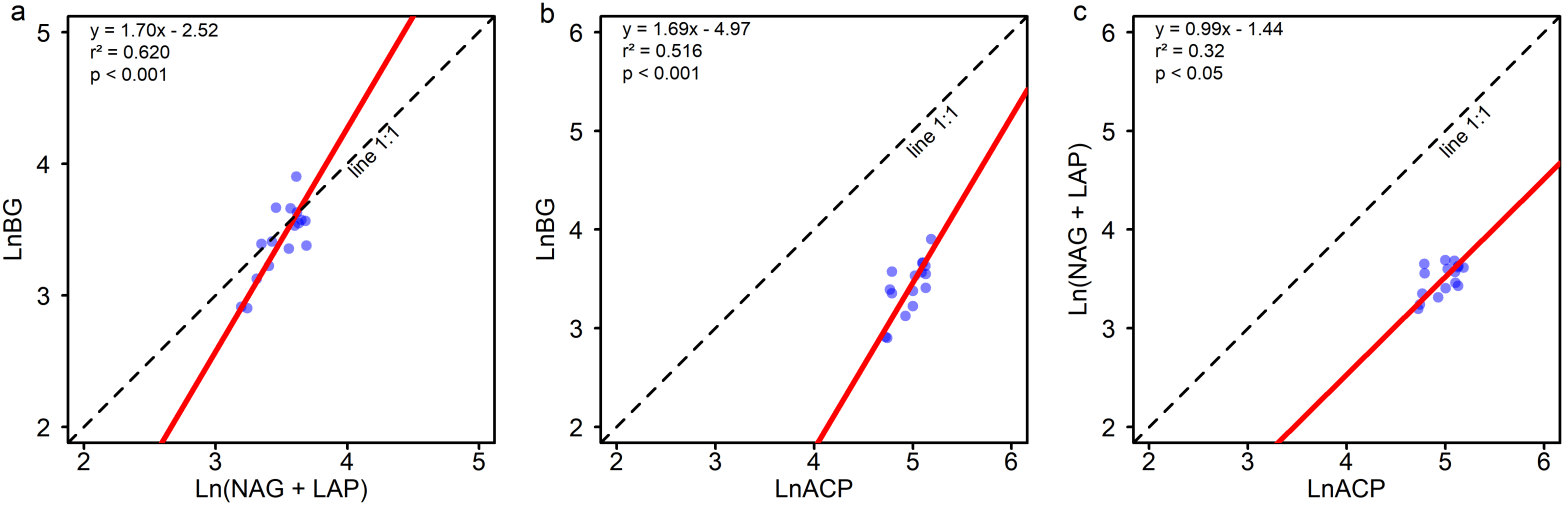
Standardized major axis (SMA) regression analysis of soil C, N, and P-acquiring enzyme activities. **(a)** BG ~ (NAG + LAP); **(b)** BG ~ ACP; and **(c)** (NAG + LAP) ~ ACP. BG, β-1,4glucosidase; NAG, β-1,4-N-acetylglucosaminidase; LAP, L-leucine aminopeptidase; ACP, acid phosphatase. Line 1:1 means the linear regression equation with a slope of 1 and an intercept of 0.

Soil carbon acquisition enzyme (BG) activity increased with the level of Moso bamboo invasion (**Figure 1a**). BG enzyme activity in the later stages of invasion (LM, HM, MB groups) is significantly (P ≤ 0.05) higher than in the early stage (BF group). The activities of nitrogen acquisition enzymes (NAG+LAP) and phosphorus acquisition enzyme (ACP) showed a pattern of MB>LM>HM>BF (**Figures 1b, c**). NAG+LAP enzyme activities in MB and LM were significantly (P ≤ 0.05) higher than in BF, while ACP enzyme activity showed significant differences between MB and BF. The soil enzyme stoichiometric ratios C:N_EEA_, C:P_EEA_, and N:P_EEA_ ranged from 0.9-1.08, 0.61-0.75, and 0.67-0.7, respectively (**Figures 1d–f**). All three enzyme ratios showed increasing trends with greater Moso bamboo invasion. C:P_EEA_ in LM, HM, and MB was significantly (P ≤ 0.05) higher than in the early stage (BF group). Standardized major axis SMA regression analysis indicates (**Figure 2**) a high correlation among enzymes responsible for C, N, and P acquisition (P<0.001). The SMA slopes for C:N_EEA_, C:P_EEA_, and N:P_EEA_ are 1.7, 1.69, and 0.99, respectively, with corresponding intercepts of -2.52, -4.97, and -1.44. Most of the C:N_EEA_ points fall along the 1:1 slope line, whereas the results for C:P_EEA_ and N:P_EEA_ deviate significantly (P ≤ 0.05) from the 1:1 slope line. Overall, the C:N_EEA_, C:P_EEA_, and N:P_EEA_ ratios deviate significantly (P ≤ 0.05) from 1:1:1.

### 3.4 Changes in soil microbial nutrient limitation

**Figure 3:**
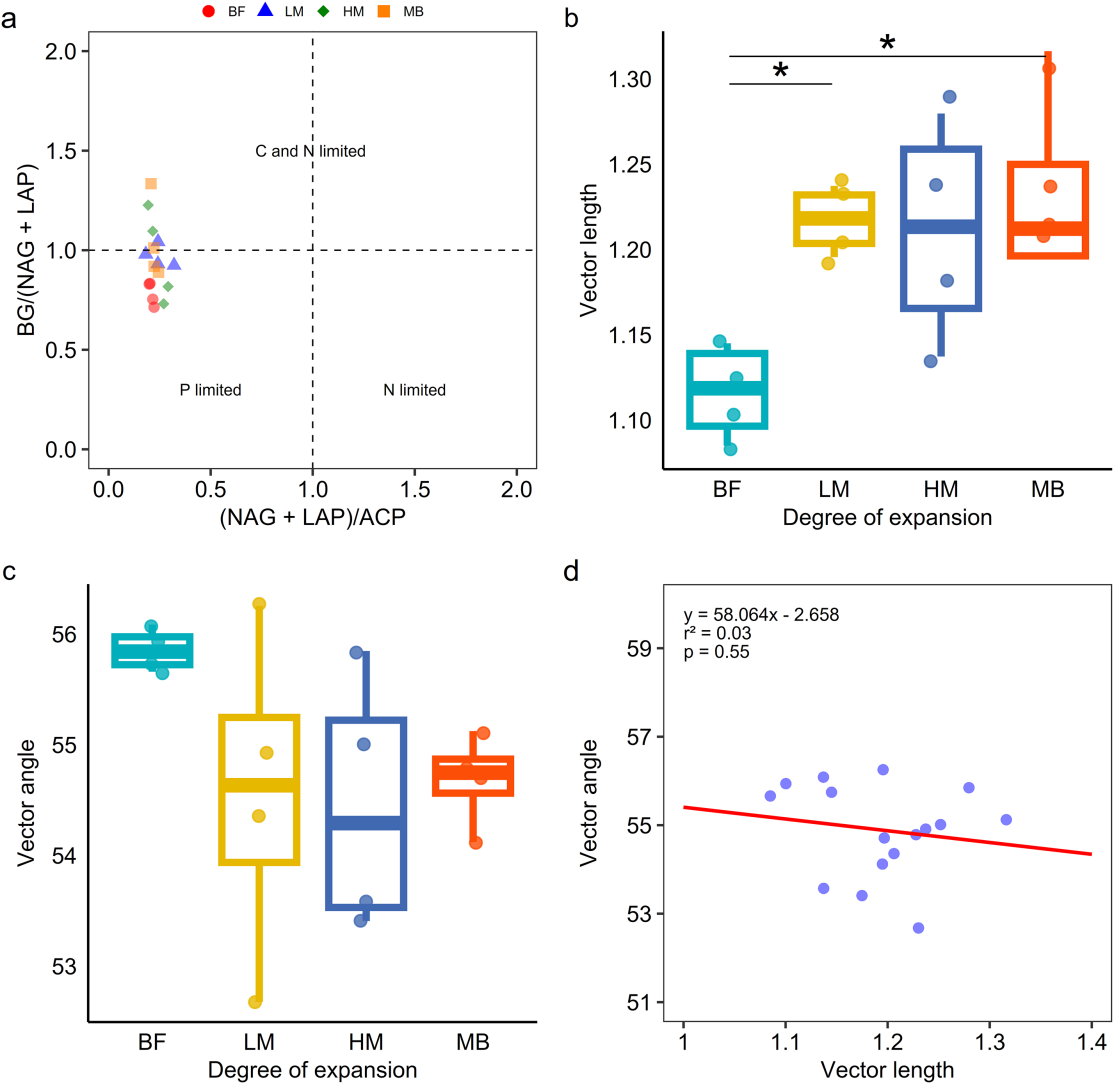
The pattern of microbial resource nutrient identified by the scatter plot of soil enzymatic stoichiometry **(a)**. Vector length response to Moso bamboo invasion progressed **(b)**. Vector angle response to Moso bamboo invasion progressed **(c)**, data were presented as mean ± SD (N = 4, * p ≤ 0.05). The relationships of Vector length (microbial C limitation) and Vector angle (microbial N/P limitation) **(d)**.

In the enzyme ratio scatter plot (**Figure 3a**), soil from the broadleaf forest (BF) was located in the phosphorus-limited quadrant. In contrast, soils from LM, HM, and MB were distributed in both the phosphorus-limited and carbon-nitrogen-limited quadrants. Vector feature analysis of enzyme stoichiometry (**Figures 3b, c**) revealed that vector lengths significantly (P ≤ 0.05) increased with the degree of Moso bamboo invasion, indicating that Moso bamboo invasion significantly (P ≤ 0.05) enhanced soil carbon limitation. The vector angles for BF, LM, HM, and MB were all greater than 45°, suggesting that soil microorganisms were phosphorus-limited. As the degree of invasion increased, the vector angles decreased, indicating a partial alleviation of phosphorus limitation for soil microorganisms. SMA regression analysis showed no significant correlation between vector length and angle (P = 0.55) (**Figure 3d**).

### 3.5 Factors affecting microbial nutrient limitation

**Figure 4:**
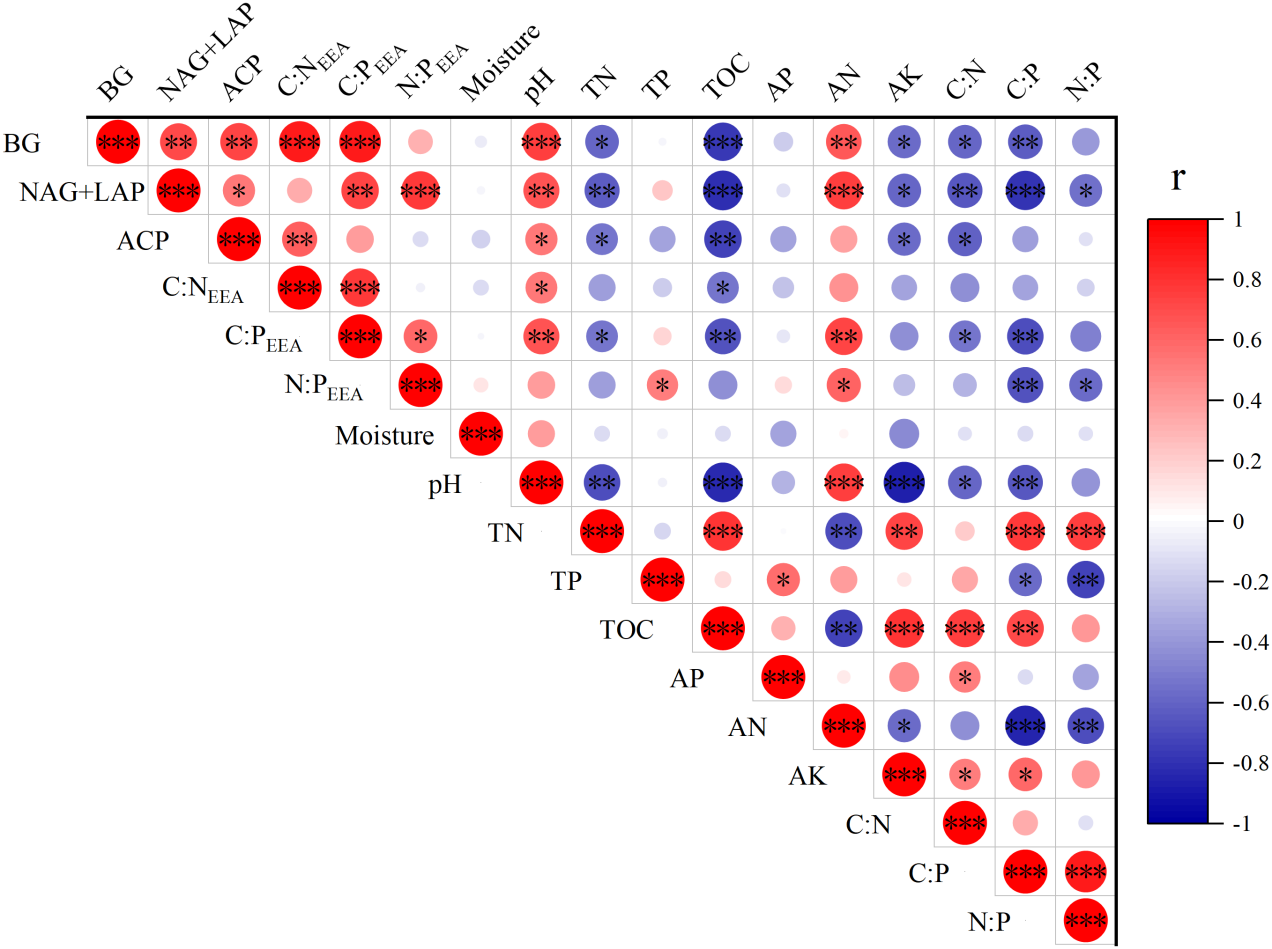
Relationships between the soil properties and soil enzymatic activities (*P≤ 0.05, **P≤ 0.01, ***P≤ 0.001). The red and blue colors indicate positive and negative correlations respectively, with color intensity proportional to the correlation coefficients (r). The r values range from -1 to 1 as shown in the legend bar.

**Figure 5:**
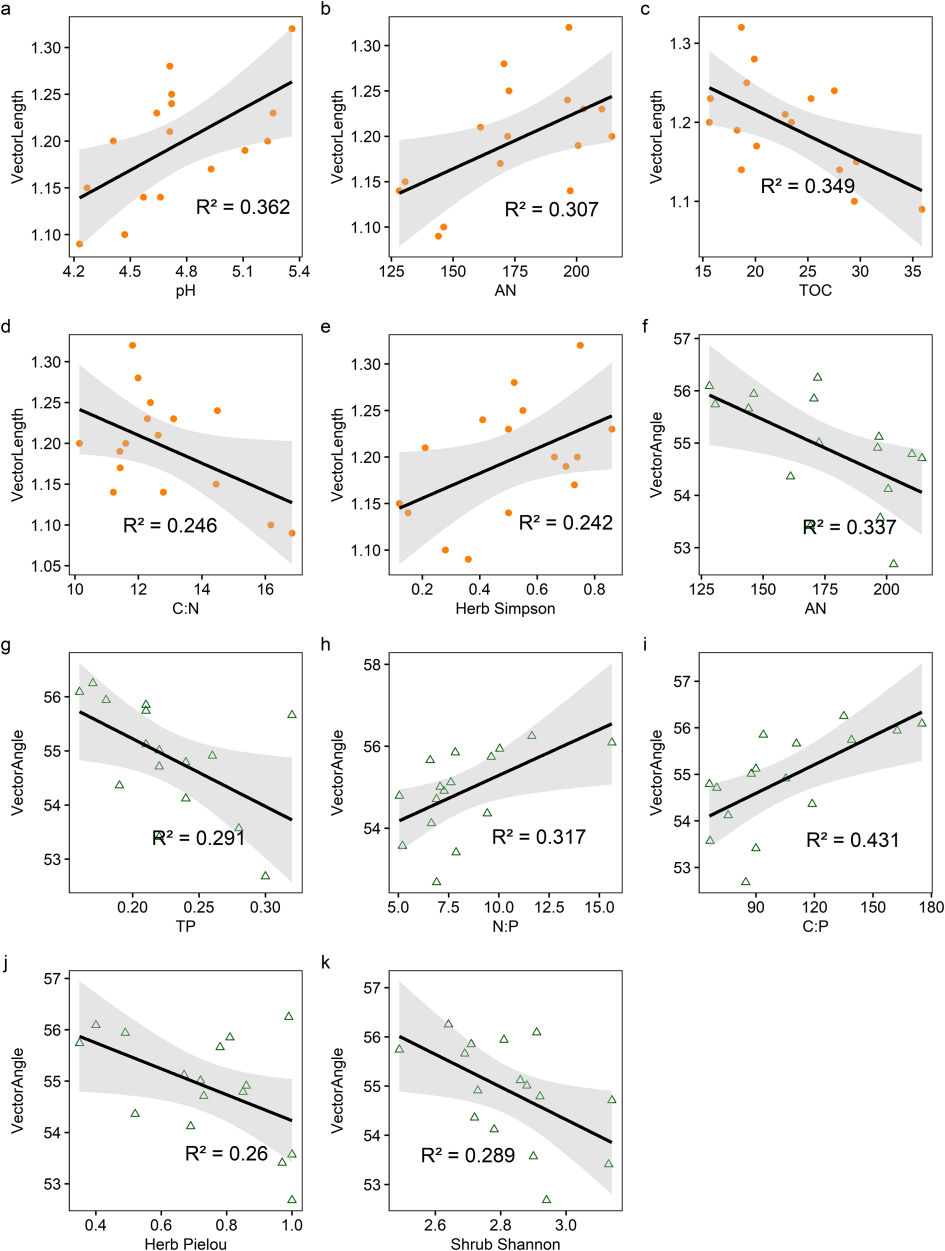
The relationship among soil physicochemical properties, species diversity indices, and vector characteristics **(a-k)**. vector length and vector angle are represented by different shapes and colors. The orange circle and green triangle represent vector length and vector angle, respectively. The solid lines represent the linear regressions, while the gray shading indicates the 95% confidence interval. All picture were significant (p ≤ 0.05).For exact statistical values, see **Supplementary Table S2** and **S3**.

**Figure 6:**
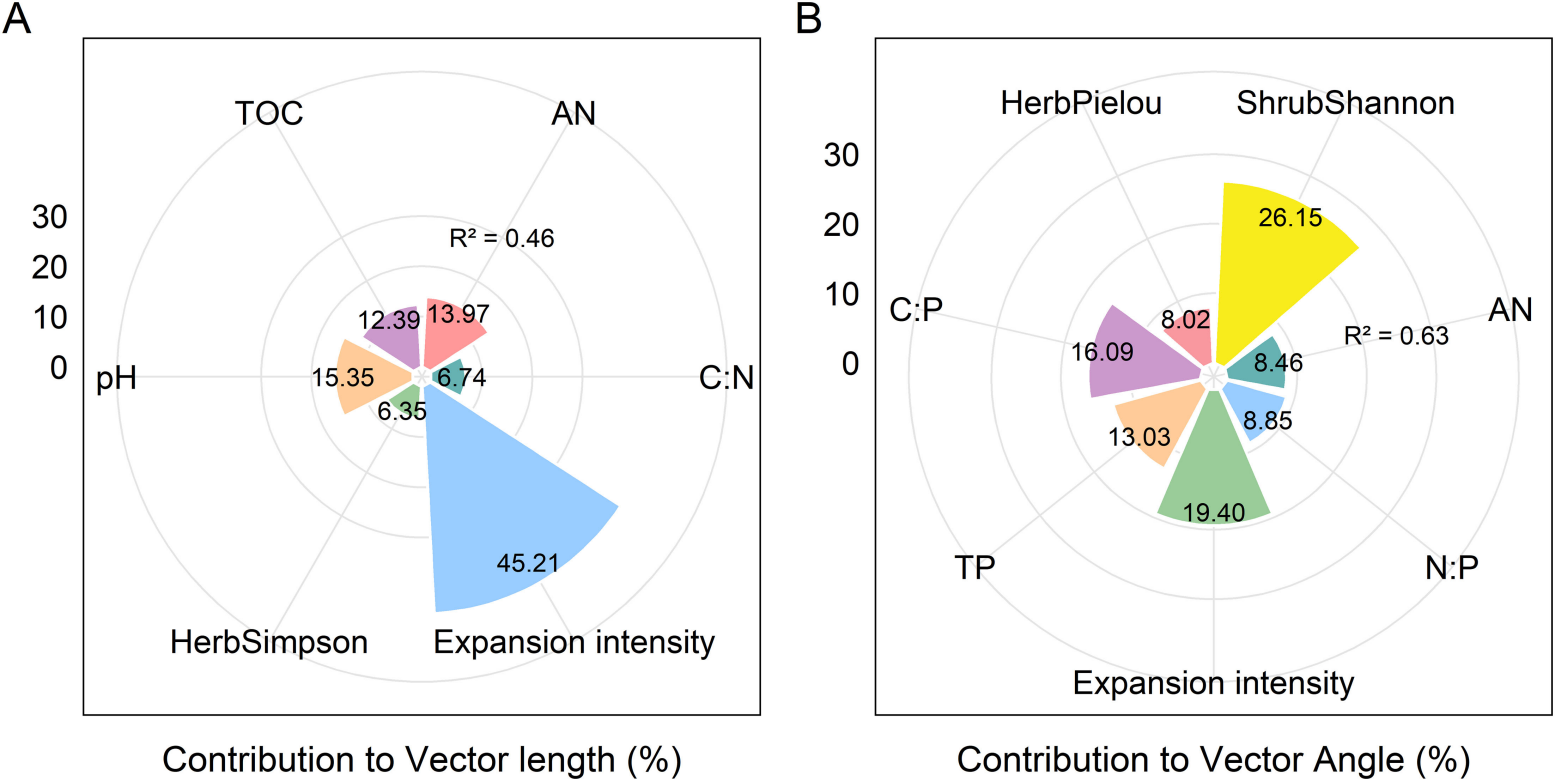
Variance partitioning of microorganism nutrient limitations moments. Contribution of soil physicochemical properties and species diversity indices to vector length (microbial C limitation) **(A)**. Contribution of soil physicochemical properties and species diversity indices to vector Angle (microbial N/P limitation) **(B)**.

The activities of BG, NAG + LAP and soil enzymatic stoichiometric ratios (C:N_EEA_, C:P_EEA_, and N:P_EEA_) showed significant (P ≤ 0.05) positive correlations with soil pH and AN content (P<0.05), but significant (P ≤ 0.05) negative correlations with TN, TOC, AK content, C:N, and C:P. Only N:P_EEA_ exhibited a significant (P ≤ 0.05) positive correlation with TP content, while NAG + LAP and N:P_EEA_ showed significant (P ≤ 0.05) negative correlations with the soil N:P ratio (**Figure 4**). We found strong relationships between microbial nutrient limitation and both soil nutrient characteristics and community structural features (**Figure 5**, **Supplementary Table S2**, **3**.). Specifically, soil AN, TP content, C:P, and shrub Pielou index showed significant (P ≤ 0.05) negative correlations with microbial phosphorus limitation, while soil N:P ratio and herbaceous Shannon index demonstrated significant (P ≤ 0.05) positive correlations with microbial phosphorus limitation (**Figure 5**). Soil pH, AN content, and herbaceous Simpson index exhibited significant (P ≤ 0.05) positive correlations with microbial carbon limitation, while soil TOC and C:N showed significant (P ≤ 0.05) negative correlations with microbial carbon limitation (**Figure 5**). Overall, the shrub Shannon index emerged as the primary factor affecting enzyme activity vector length, while the degree of Moso bamboo expansion was the most important factor influencing enzyme activity vector angle (**Figure 6**).

## 4 Discussion

### 4.1 Impacts of bamboo invasion on forest community and soil properties

Invasions by either non-native or native plant species can substantially alter community species composition (Nackley et al., 2017; Ouyang et al., 2022). Our study revealed that tree-layer species diversity declined significantly as Moso bamboo invasion intensity increased (**Table 1**). Moso bamboo’s rapid growth and robust clonal reproduction allow it to outcompete other species for space and resources, thereby inhibiting broadleaf saplings and gradually eliminating certain tree species (Tian et al., 2020). Furthermore, Moso bamboo’s extensive root system efficiently extracts soil nutrients and water, intensifying competitive pressure on neighboring vegetation and severely limiting tree seedling establishment and survival (Fang et al., 2022). Interestingly, species diversity in the shrub and herb layers exhibited an increasing trend during the early and middle stages of bamboo expansion, with particularly pronounced increases in the herb layer (**Table 1**). This phenomenon may be attributed to bamboo invasion altering the understory microenvironment, creating more niches for shade-tolerant and ruderal herbaceous species (Lacerda and Kellermann, 2019). In the early invasion stage (LM plots), partial thinning of the broadleaf canopy by bamboo allows more light to reach the forest floor, which can promote the growth of shrubs and herbs that were previously light-limited. We observed an increase in sun-loving shrub species and groundcover plants under these conditions. However, in later invasion stages (MB plots), understory diversity did not continue to increase and sometimes declined in quality, becoming dominated by a few hardy species. Thus, bamboo invasion initially creates a more shrub-dominated understory after the loss of overstory trees, but if bamboo stands become extremely dense, even shrub diversity may eventually decline due to bamboo’s thick litter and aggressive root competition. Our LM and HM plots likely represent a transient peak in understory diversity, which could diminish if bamboo continues to accumulate biomass unchecked. Notably, this heightened understory diversity should not be interpreted as improved ecosystem health; instead, it may reflect underlying community instability and rapid species turnover (Tilman, 1999).

Soil physicochemical changes under bamboo invasion further illustrate its ecosystem impacts. In our study, greater bamboo invasion corresponded with significant decreases in soil TOC, TN, and AK contents, indicating a trend of soil nutrient depletion (**Figure 2**). These reductions are mainly attributable to bamboo’s extensive biomass production and consequent rapid nutrient uptake, combined with its slowly decomposing litter which impedes nutrient return to the soil (Kaushal et al., 2020; Tian et al., 2020). The net effect is a bamboo-invaded soil with diminished fertility and altered chemistry. Such nutrient-poor conditions can further hinder the re-establishment of broadleaf trees, reinforcing bamboo dominance through a positive feedback. Our results concur with those of Chen et al. (2024), who found reduced soil quality in oak forests invaded by bamboo. Moreover, soil pH showed a positive correlation with bamboo invasion intensity (**Table 2**), increasing from the broadleaf forest to bamboo stands. This pH shift can be attributed to the chemical composition of bamboo litter: its high carbon-to-nitrogen ratio and low nutrient content may reduce soil acidity (Chang et al., 2019). Changes in soil pH can, in turn, affect microbial community composition and function, thereby influencing nutrient cycling processes (Guo et al., 2016). In summary, by altering the forest structure and depleting soil organic matter and nutrients, Moso bamboo acts as an ecosystem driver in these forests. It not only replaces native trees but also engineers the soil environment, setting the stage for subsequent microbial responses.

### 4.2 Impact of Moso bamboo invasion on soil enzyme activity and microbial nutrient limitation

Soil enzyme activity serves as a direct indicator of microbial nutrient cycling processes. Our results demonstrated that the activities of carbon-acquisition (BG), nitrogen-acquisition (NAG+LAP), and phosphorus-acquisition (ACP) enzymes all increased with the degree of bamboo invasion (**Figure 1**). This indicates that soil microorganisms enhanced their efforts to acquire C, N, and P during bamboo expansion. When soil resources become insufficient to meet microbial metabolic demands, microorganisms respond by secreting more enzymes to alleviate nutrient deficiencies while maintaining their growth and reproductive functions (Baek et al., 2024). The observed changes in soil nutrient levels are consistent with this pattern: bamboo invasion led to declining soil TOC, TN, AP, AK, and C:N, C:P, N:P ratios (**Table 2**), signaling both imbalanced nutrient stoichiometry and acute nutrient scarcity (Zhang, 2023).

In essence, as resources became limiting, the microbes “worked harder” by expending more energy on enzyme production to scavenge those scarce nutrients. Bamboo invasion deprived the soil of easily available carbon and nitrogen, so microbes ramped up C- and N-degrading enzyme activities to meet their nutritional needs (Liu et al., 2023). The particularly large increase in BG activity with invasion underscores that carbon limitation became a primary constraint for the microbial community. BG targets cellulose and other polysaccharides; in native broadleaf forest, continuous leaf and twig litter inputs provide carbon substrates, whereas in bamboo stands, litter inputs may be reduced or consist of more recalcitrant material, leading to periods of carbon scarcity. The high BG activity we observed suggests that microbes in bamboo-invaded soil are actively trying to decompose whatever carbon sources are available to obtain energy. This could potentially accelerate the loss of soil carbon — a worrisome positive feedback, whereby bamboo reduces carbon inputs and microbes then deplete existing soil carbon faster via elevated enzyme activity (Chang and Chiu, 2015). NAG and LAP activities also increased, though to a slightly lesser extent than BG, indicating that microbes were also experiencing N limitation, but carbon was relatively more limiting (Sardar et al., 2023). ACP activity roughly doubled even as available P declined by ~40%, which is consistent with microbes initially facing P limitation and thus producing much more phosphatase to scavenge P as it became scarce (Wu et al., 2018). However, because P limitation was somewhat alleviated in a relative sense under bamboo (as indicated by the vector angle analysis), the large increase in ACP might also be partly due to the pH increase, since phosphatase activity tends to rise as very acidic soils become less acidic. It is important to note that a reduction in relative P limitation does not mean P became abundant; rather, it means that N (and C) became even more limiting compared to P. Overall, the microbial community in bamboo-invaded soil exhibited a clear stress response of increased enzyme production to acquire carbon and other nutrients, supporting our first hypothesis: microbial carbon limitation intensified and the balance of N vs. P limitation shifted under bamboo invasion.

SMA regression of enzyme activities confirmed that the ratios of C-, N-, and P-acquiring enzymes in bamboo-invaded soils deviated from the typical global average of 1:1:1 (Sinsabaugh et al., 2008). In particular, the SMA slopes for BG:(NAG+LAP) and BG: ACP were significantly different from 1 (**Figure 2**), indicating disproportionate shifts in microbial allocation toward carbon acquisition. This suggests that bamboo expansion has altered the ecosystem’s functional stoichiometry, leading to changes in the proportional allocation of microbial effort among different nutrient acquisition pathways (Yang et al., 2020). Enzyme stoichiometry and vector analyses further demonstrated significant changes in microbial nutrient limitation status: carbon limitation increased markedly with progressing bamboo invasion (**Figure 3b**). This intensified microbial C limitation is one of the clearest outcomes of our study, meaning that microbial activity in bamboo-dominated stands is now more constrained by carbon scarcity than in the native broadleaf forest. Beyond reduced litter quantity, bamboo litter quality likely intensifies microbial C-limitation. With Moso bamboo expansion, the lignin content of the litter layer increases and the lignin:N ratio tends to be higher, while overall litter nutrient stocks decline—conditions that typically favor recalcitrant SOC formation and constrain microbial access to labile C. In parallel, bamboo is a strong silicon accumulator, and phytolith-occluded carbon (PhytOC) embedded in silica matrices is highly resistant to decomposition, further shifting the SOC pool toward forms that are less microbially available. These litter-chemistry and silica-stabilization pathways provide a mechanistic link from bamboo dominance to elevated BG activity and C-acquiring enzyme investment, consistent with our eco-enzymatic vectors (Lv et al., 2020; Manzoni et al., 2021; Chen et al., 2024). In the broadleaf forest, despite inherently low phosphorus availability, continuous carbon inputs (from litterfall and root exudates) likely prevented severe carbon limitation; thus, phosphorus was the primary limiting nutrient for microbes (as is common in old, weathered soils). Once bamboo came to dominate, the reduction in carbon inputs (and possibly faster carbon turnover) made carbon the scarcest resource relative to microbial demand (Kamalanathan et al., 2020). While soil microbes in our study region were strongly phosphorus-limited under broadleaf forest, the shift to bamboo forest partially alleviated that P limitation, as evidenced by the decrease in enzyme vector angle (though all angles remained >45°). This partial alleviation of P stress may be attributed to bamboo’s nitrophilic nature and the relatively sufficient soil N in these stands (**Table 1**), which reduced microbial dependence on phosphorus and allowed more efficient phosphorus utilization in bamboo soils (Li et al., 2017). Furthermore community shifts from ECM-associated trees to AM-dominant vegetation under bamboo expansion may alter P-acquisition pathways toward direct soil Pi uptake by AMF, reducing the need for ECM-mediated organic P mining and thereby raising plant-available P (Dong et al., 2021; Pantigoso et al., 2023). The weak correlation we found between enzyme vector length and angle (**Figure 3d**) supports the interpretation that changes in overall limitation intensity (C limitation) were largely independent of changes in the type of limitation (N vs P). However, it is important to stress that the alleviation of P limitation was only partial: phosphorus is still not abundant in bamboo-invaded soil (AP was lower in absolute terms than in BF). The vector angle in the MB stage (~50°) suggests a slight shift toward N limitation dominance, but not an extreme one—essentially indicating that microbes became co-limited by C and N, with P remaining a secondary constraint. In such a more balanced co-limited scenario, alleviation of one nutrient limitation (here P) can help microbes better utilize another nutrient (N), potentially leading to more efficient decomposition when no single element is overwhelmingly limiting.

### 4.3 Main factors influencing microbial nutrient limitation

Our findings revealed strong interconnections between microbial nutrient limitation status, soil nutrient conditions, and plant community structure. Soil nutrient status directly influences microbial enzyme allocation strategies. In our study, BG and NAG+LAP activities, along with the enzyme activity ratios (C:NEEA, C:PEEA, N:PEEA), were positively correlated with soil pH and AN content (Yao et al., 2023), indicating that higher pH and greater inorganic N availability stimulate microbes to secrete more enzymes to meet their nutrient demands. Conversely, these enzyme metrics showed negative correlations with soil TOC, TN, AK, C:N, and C:P (**Figure 4**), suggesting that in soils rich in organic matter or with more balanced C and nutrient ratios, microbes did not need to increase enzyme production (Fujita et al., 2019).

Regression analyses further highlighted that microbial carbon limitation (vector length) was positively associated with soil pH, AN, and herb-layer Simpson diversity, but negatively associated with soil TOC and C:N. This implies that abundant organic matter or optimal C:N ratios reduce microbial carbon stress. Moreover, a higher diversity in the shrub layer can contribute more organic inputs to the soil, which likely helps alleviate microbial carbon limitation (Song et al., 2021). In terms of phosphorus limitation (vector angle), soil AN, TP, C:P, and shrub-layer Pielou’s evenness were significantly negatively correlated with microbial P limitation, whereas soil N:P and herb-layer Shannon diversity were positively correlated (**Figure 5**). These patterns suggest that when available N and P are relatively sufficient or the plant community is more even (less dominance), microbes experience less P limitation; in contrast, environments with high soil N:P ratios or very diverse herb layers may impose greater P demands on microbes, thereby increasing microbial P limitation (Wu et al., 2022). In summary, both soil nutrient availability and plant community diversity significantly influence microbial nutrient limitation status. Notably, the shrub layer Shannon diversity index emerged as the primary factor associated with microbial carbon limitation (**Figure 6a**), whereas the degree of bamboo expansion was the most influential factor on microbial phosphorus limitation (**Figure 6b**). This likely occurs because bamboo expansion dramatically alters community structure and litter dynamics, which in turn affect soil organic matter inputs and microbial nutrient demands (Fang et al., 2022).

We found that shifts in microbial nutrient limitation can, in turn, alter nutrient cycling pathways in the ecosystem. Under the broadleaf forest (P-limited microbes), soil microbes likely immobilize available nitrogen (because N was relatively abundant compared to P), resulting in slow nitrogen release, while any available phosphorus is immediately taken up. This could lead the system to accumulate N in organic forms (since microbes cannot mineralize N efficiently without adequate P) and to conserve P tightly. Under the bamboo stand (microbes limited by C and N), microbes will mineralize whatever phosphorus they acquire (since P is not limiting, they do not need to retain it), but will immobilize carbon and nitrogen whenever possible (Baek et al., 2024). This situation could result in faster P turnover—potentially more P leaching or uptake by bamboo as P is freed—and a lower carbon use efficiency by microbes (more CO_2_ released per unit C assimilated). In other words, the bamboo-invaded ecosystem may become leaky for P but tight for N. Bamboo might inadvertently allow some P to remain unused if it does not require as much P as the previous broadleaf system; that phosphorus could accumulate in forms unavailable to biota or be lost via leaching in heavy rainfall (though given the low absolute P levels, leaching losses are likely minimal) (Zhang, 2023). It is informative to compare our native bamboo invasion scenario to more commonly studied exotic plant invasions. Many exotic invaders (e.g., Melastoma or Lantana in tropical regions, Fallopia in temperate regions) can also increase soil pH or alter litter quality. Some, especially N-fixing invaders, alleviate nitrogen limitation but exacerbate phosphorus limitation in soils (Thorpe et al., 2013; Zhang et al., 2024). Our case is somewhat the opposite: a non-N-fixing invader (bamboo) alleviated P limitation but intensified N limitation. A meta-analysis by Jo et al. (2017) found that plant invasions often increase soil N availability via accelerated N cycling; in contrast, our results showed a decrease in N availability under bamboo. The difference likely arises because bamboo does not introduce new N to the system (no N-fixation) and because it is a native species expansion rather than an exotic with novel nutrient traits (Jo et al., 2017; Zhang and Suseela, 2021). This comparison highlights that each invasion can have unique nutrient cycling consequences.

### 4.4 Linking plant diversity and soil changes to microbial limitations

Our results underscore that the changes in microbial nutrient limitation are closely linked with the changes in plant community and soil properties brought about by bamboo invasion. Here, we discuss the key drivers in more detail.

We found that higher shrub-layer diversity was strongly correlated with lower microbial carbon limitation. Shrubs likely provide alternative carbon sources that help sustain microbial communities. In the broadleaf forest (BF), a diverse canopy and shrub assemblage produces a mosaic of litter types, supporting a more balanced “diet” for microbes. Bamboo invasion simplifies the litter input to mostly bamboo leaves, but the presence of other understory plants in invaded plots mitigates this effect. Additionally, shrubs can access nutrients from deeper soil layers or different nutrient pools and then redistribute them to surface soil via litterfall, effectively “pumping” nutrients that bamboo might not access and making them available to microbes (Jobbágy and Jackson, 2004; Zuo et al., 2023). For example, a nitrogen-fixing shrub would add nitrogen to the system, and a shrub with litter high in polyphenols could slow decomposition, providing a slow-release carbon source throughout the year. In the absence of shrubs, bamboo litter might decompose quickly and then leave the soil with periods of carbon scarcity. This points to a management insight: maintaining or promoting native understory vegetation in bamboo-invaded areas might buffer soil processes. While this may not stop bamboo spread, it could prevent the functional collapse of soil nutrient cycling. Indeed, studies on mixed bamboo–broadleaf stands have found that mixing broadleaf trees with bamboo improves soil nutrient status and microbial diversity (Yang et al., 2023).

Bamboo’s strong preference for nitrogen was highlighted by Li et al. (2017), and our findings provide evidence of this as well: the bamboo-invaded plots had relatively lower inorganic N levels, and microbial communities shifted toward greater N limitation. Bamboo, like many grasses, is very efficient at N uptake. In mixed stands, bamboo likely monopolizes a large share of soil N (especially during peak growth when new culms and leaves are formed), which can starve both microbes and other plants of nitrogen. This explains why we observed consistently high enzyme vector angles (indicating N limitation) in bamboo stands—soil microbes simply could not obtain enough nitrogen because bamboo was extracting it, and also because there was less nitrogen being returned via litter (broadleaf litter often has higher N content than bamboo litter). Thus, bamboo’s nutrient uptake strategy (rapid, shallow N uptake) directly forces soil microbes to adjust by investing more in N-acquisition enzymes relative to P-acquisition.

Overall, this study highlights the tight coupling between plant community composition and soil microbial function in the context of invasion. Even a native species like Moso bamboo, when behaving invasively, can act as an ecosystem engineer that reconfigures fundamental nutrient limitations, with far-reaching effects on decomposition processes and nutrient feedbacks. Our findings provide insight for invasion ecology theory and practical forest management alike. Protecting ecosystem health in the face of bamboo invasion will require an integrated approach that addresses both the symptom (unchecked bamboo spread) and the underlying drivers (altered soil conditions and microbial nutrient cycling). Such measures will help conserve the biodiversity and sustain the soil resources of subtropical forests over the long term.

## 5 Limitations and future directions

While our space-for-time study reveals clear patterns, it has inherent limitations. We inferred temporal changes from spatial differences, assuming the BF plots represent the pre-invasion state of the current MB plots. It is possible that the plots had some differences even before bamboo invasion. We attempted to minimize this by careful transect selection, but we cannot be certain that invasion is the sole cause of all observed differences. Long-term monitoring of permanent plots as bamboo invades would be ideal to confirm these results. For instance, tracking a broadleaf forest plot over time as bamboo density increases would directly show how soil carbon and enzyme activities change, strengthening causal inference.

We also did not measure microbial biomass, community composition, or functional gene profiles, which limits our understanding of the underlying mechanisms. Metagenomic or metatranscriptomic analyses could identify whether certain microbial groups (e.g., those efficient at P acquisition or those with high C demand metabolisms) become more abundant under bamboo. Such analyses could also reveal whether the genes encoding the enzymes we measured are present in higher copy numbers (indicating a change in genetic potential) or are more actively expressed (indicating an acute response) in invaded soils (Nwachukwu and Babalola, 2022).

Our study opens several avenues for further research. First, microbial community analysis: future work should identify which microbial taxa and functional groups are most affected by bamboo invasion. High-throughput DNA/RNA sequencing could reveal shifts in community composition and functional gene abundance. For example, do bamboo-invaded soils harbor fewer ectomycorrhizal fungi and more opportunistic saprotrophic fungi or bacteria? Such information would help link changes in community structure to changes in enzyme activity (Nunez-Mir and McCary, 2024). Second, long-term experiments and chronosequence validation: establishing permanent plots to observe the invasion process (and any recovery if bamboo is removed) over time would provide dynamic data to validate our space-for-time assumptions. If possible, combining current invaded vs. intact stand comparisons with historical data or dendrochronological analysis might help infer past changes. Third, investigating plant–soil feedbacks on forest regeneration: it would be insightful to test how the altered soil conditions under bamboo affect the germination and growth of native tree seedlings. Bamboo-invaded soil, with its low nutrient status, might impair broadleaf seedling establishment, creating a negative feedback that keeps bamboo dominant. Experiments planting tree seedlings in soil collected from BF vs. MB plots could reveal growth differences. If seedlings grow poorly in MB soil, additional tests (e.g., adding nutrients or sterilizing soil) could determine whether the limitation is due to nutrient deficiency (reversible with fertilization) or adverse soil biota (possibly accumulation of pathogens in bamboo soil). This line of inquiry touches on the enemy-release versus enemy-accumulation hypotheses, as bamboo might cultivate soil biota that inhibit other plants (Li et al., 2024).

Given the increasing occurrence of abandoned Moso bamboo forests invading adjacent woodlands, our results and other studies suggest several management strategies:

1. Regular monitoring and early intervention: Continuously monitor forest edges where bamboo plantations border native forests. Early detection of bamboo spread (for example, via remote sensing of canopy changes or routine ground surveys) allows timely intervention. If bamboo culms are observed establishing in a native forest understory, managers should act quickly to cut those culms and remove or sever the connecting rhizomes. Physical containment measures such as underground rhizome barriers or trenching around bamboo stands can prevent further spread of rhizomes. Local policies might also encourage or require bamboo growers to contain their stands (similar to regulations in some areas for containing running bamboo). Early intervention is much more effective and feasible than eliminating an extensive, established bamboo invasion.

2. Soil amendments and fertilization: In severely invaded stands where soil organic matter is depleted, adding organic amendments could help restore soil fertility and support native plant recovery. For instance, applying composted broadleaf litter or manure can increase soil carbon and nutrient levels, giving soil microbes and native plants a needed boost. This treatment could alleviate the microbial carbon starvation observed under bamboo and enhance nutrient mineralization for regenerating vegetation. In bamboo agroforestry, practices like mulching with rice straw or legume residues are used to maintain soil fertility (Gai et al., 2021); similar approaches could be adapted for ecological restoration in invaded forests. Additionally, if soil tests indicate critically low levels of N or P, targeted fertilization might be considered to rebalance nutrients (e.g., adding nitrogen to stimulate broadleaf regrowth, or phosphorus if it is severely limiting tree establishment). Any fertilization should be judicious and likely confined to active restoration sites, as fertilizing natural forests is not common practice.

3. Maintaining landscape-level diversity: At a landscape scale, land-use planning should avoid large contiguous monocultures of bamboo. Instead, a mosaic landscape with mixed native forests and bamboo stands is preferable. If bamboo plantations are being abandoned, managers should either continue low-intensity harvests or thinning to keep bamboo in check, or actively facilitate the transition of those areas back to native forest (for example, through planting native tree species or encouraging natural succession). Government incentives could support the restoration of abandoned bamboo fields to native forest as a means to conserve biodiversity and ecosystem services. Public education is also important: local communities may view dense bamboo forests as benign or even aesthetically pleasing, not realizing they can indicate declining soil health and biodiversity loss. Raising awareness about the hidden impacts of uncontrolled bamboo spread can build support for management interventions.

4. Monitoring soil health indicators: Incorporating soil health metrics into invasion monitoring can provide early warning signs of ecosystem degradation. Managers could track soil microbial indicators such as enzyme activity ratios (like BG: NAG or C:N acquisition ratios) or simpler measures like soil respiration and microbial biomass C/N ratio. For example, a sharp drop in soil C:N ratio or a disproportionately high β-glucosidase (C-degrading) to N-acquiring enzyme activity ratio in the forest floor could signal deteriorating soil conditions due to bamboo. These soil indicators can complement vegetation surveys to assess the impact of invasion and to gauge the success of management actions over time.

## 6 Conclusion

Our findings demonstrate that bamboo expansion significantly alters forest structure and soil resource availability, which in turn drives pronounced shifts in microbial nutrient acquisition strategies. Specifically, bamboo invasion caused a shift from a predominantly P-limited microbial regime in the native broadleaf forest to a regime more limited by C and N in the bamboo-invaded forest. Microbes in bamboo stands were forced to invest more in carbon-acquiring enzymes due to reduced organic carbon inputs, intensifying microbial carbon limitation. At the same time, phosphorus scarcity was somewhat alleviated (likely due to increased soil pH and reduced competition for P), causing the primary microbial nutrient limitation to pivot toward nitrogen. These microbial nutrient limitation changes were closely linked to changes in the plant community and soil properties: the loss of tree diversity and reduced litter inputs under bamboo created carbon-poor, nitrogen-competitive soil conditions, while remnants of shrub diversity in the understory helped buffer microbes against the most extreme nutrient limitations.

## Data Availability

The original contributions presented in the study are included in the article/**Supplementary Material**. Further inquiries can be directed to the corresponding author.
