# Supplementary material for: Moso bamboo invasion alters soil microbial nutrient limitation by modifying plant diversity and soil nutrient cycling in subtropical forest
Source: Front Plant Sci. 2025 Oct 30;16:1549018. doi: 10.3389/fpls.2025.1549018 (PMC12614466; doi:10.3389/fpls.2025.1549018)
Supplement: Supplementary file 1 [file Table1.docx]

**Supplementary materials**

**Table S1 The proportion of Moso bamboo and the number of plants at different layers in 16 plots**

| Forest type | Plot | Bamboo  proportion(%) | Stems of  Moso bamboo | Stems of  trees | Total stems of  trees | Stems of  shrubs | Stems of  herbs |
| --- | --- | --- | --- | --- | --- | --- | --- |
| BF | BF1 | 0 | 0 | 158 | 158 | 183 | 15 |
|  | BF2 | 0 | 0 | 129 | 129 | 80 | 13 |
|  | BF3 | 0 | 0 | 114 | 114 | 155 | 32 |
|  | BF4 | 0 | 0 | 70 | 70 | 145 | 50 |
| LM | LM1 | 28 | 53 | 134 | 187 | 204 | 8 |
|  | LM2 | 34 | 53 | 104 | 157 | 158 | 6 |
|  | LM3 | 26 | 43 | 124 | 167 | 180 | 7 |
|  | LM4 | 22 | 30 | 106 | 136 | 136 | 17 |
| HM | HM1 | 62 | 109 | 67 | 176 | 223 | 11 |
|  | HM2 | 66 | 80 | 42 | 122 | 205 | 4 |
|  | HM3 | 61 | 60 | 38 | 98 | 245 | 25 |
|  | HM4 | 75 | 86 | 28 | 114 | 191 | 20 |
| MB | MB1 | 100 | 136 | 0 | 136 | 397 | 157 |
|  | MB2 | 100 | 124 | 0 | 124 | 283 | 158 |
|  | MB3 | 100 | 117 | 0 | 117 | 240 | 101 |
|  | MB4 | 100 | 151 | 0 | 151 | 326 | 71 |

**Table S2 Results of general linear regression analysis among soil physical and chemical properties species diversity ,index and vector length**

| Group | R_squared | Slope | Intercept | P_value |
| --- | --- | --- | --- | --- |
| TP~ VectorLength | 0.0134 | -0.1625 | 1.2318 | 0.6696 |
| AP~ VectorLength | 0.0446 | -0.0096 | 1.2536 | 0.4321 |
| N:P~ VectorLength | 0.0697 | -0.0063 | 1.2468 | 0.3233 |
| AK~ VectorLength | 0.1502 | -0.0010 | 1.3025 | 0.1381 |
| TN~ VectorLength | 0.1877 | -0.0866 | 1.3498 | 0.0937 |
| C:P~ VectorLength | 0.2204 | -0.0009 | 1.2872 | 0.0666 |
| C:N~ VectorLength | 0.2576 | -0.0175 | 1.4191 | 0.0448 |
| AN~ VectorLength | 0.3153 | 0.0013 | 0.9725 | 0.0236 |
| TOC~ VectorLength | 0.3666 | -0.0066 | 1.3471 | 0.0129 |
| pH~ VectorLength | 0.3762 | 0.1127 | 0.6594 | 0.0115 |
| ShrubSimpson~ VectorLength | 0.0027 | -0.1773 | 1.3568 | 0.8479 |
| ShrubShannon~ VectorLength | 0.0120 | 0.0409 | 1.0790 | 0.6858 |
| ShrubMargalef~ VectorLength | 0.0617 | 0.0243 | 1.0644 | 0.3535 |
| HerbPielou~ VectorLength | 0.0638 | 0.0756 | 1.1389 | 0.3454 |
| ShrubPielou~ VectorLength | 0.0961 | -0.6350 | 1.7266 | 0.2425 |
| HerbMargalef~ VectorLength | 0.1805 | 0.0293 | 1.1620 | 0.1009 |
| TreeSimpson~ VectorLength | 0.2114 | -0.0831 | 1.2395 | 0.0731 |
| TreePielou~ VectorLength | 0.2126 | -0.0887 | 1.2403 | 0.0723 |
| HerbShannon~ VectorLength | 0.2152 | 0.0467 | 1.1486 | 0.0703 |
| TreeMargalef~ VectorLength | 0.2272 | -0.0227 | 1.2423 | 0.0619 |
| TreeShannon~ VectorLength | 0.2301 | -0.0328 | 1.2400 | 0.0600 |
| HerbSimpson~ VectorLength | 0.2570 | 0.1390 | 1.1249 | 0.0450 |

**Table S3 Results of general linear regression analysis among soil physical and chemical properties species diversity ,index and vector Angle**

| Group | R_squared | Slope | Intercept | P_value |
| --- | --- | --- | --- | --- |
| AP~ VectorAngle | 0.0255 | -0.1204 | 55.6252 | 0.5544 |
| AK~ VectorAngle | 0.0520 | 0.0093 | 53.8382 | 0.3956 |
| C:N~ VectorAngle | 0.0832 | 0.1650 | 52.7763 | 0.2785 |
| TN~ VectorAngle | 0.1038 | 1.0662 | 52.9796 | 0.2237 |
| pH~ VectorAngle | 0.1344 | -1.1156 | 60.1871 | 0.1625 |
| TOC~ VectorAngle | 0.1506 | 0.0703 | 53.2707 | 0.1374 |
| TP~ VectorAngle | 0.2802 | -12.3144 | 57.6965 | 0.0350 |
| N:P~ VectorAngle | 0.3160 | 0.2236 | 53.0533 | 0.0234 |
| AN~ VectorAngle | 0.3368 | -0.0216 | 58.6919 | 0.0184 |
| C:P~ VectorAngle | 0.4303 | 0.0205 | 52.7480 | 0.0058 |
| TreeMargalef~ VectorAngle | 0.0186 | 0.1076 | 54.6630 | 0.6146 |
| HerbMargalef~ VectorAngle | 0.0278 | -0.1908 | 55.1007 | 0.5370 |
| HerbShannon~ VectorAngle | 0.0408 | -0.3365 | 55.2210 | 0.4532 |
| TreeSimpson~ VectorAngle | 0.0496 | 0.6667 | 54.5289 | 0.4069 |
| TreeShannon~ VectorAngle | 0.0554 | 0.2665 | 54.5208 | 0.3803 |
| TreePielou~ VectorAngle | 0.0586 | 0.7717 | 54.4921 | 0.3662 |
| ShrubPielou~ VectorAngle | 0.0689 | -8.9014 | 62.3430 | 0.3261 |
| HerbSimpson~ VectorAngle | 0.1101 | -1.5063 | 55.6450 | 0.2094 |
| ShrubSimpson~ VectorAngle | 0.2353 | -27.3005 | 79.8510 | 0.0568 |
| ShrubMargalef~ VectorAngle | 0.2394 | -0.7938 | 59.1384 | 0.0544 |
| HerbPielou~ VectorAngle | 0.2599 | -2.5259 | 56.7557 | 0.0437 |
| ShrubShannon~ VectorAngle | 0.2900 | -3.3245 | 64.2902 | 0.0314 |
